# COVID-19-related research data availability and quality according to the FAIR principles: A meta-research study

**DOI:** 10.1371/journal.pone.0313991

**Published:** 2024-11-18

**Authors:** Ahmad Sofi-Mahmudi, Eero Raittio, Yeganeh Khazaei, Javed Ashraf, Falk Schwendicke, Sergio E. Uribe, David Moher

**Affiliations:** 1 National Pain Centre, Department of Anesthesia, McMaster University, Hamilton, ON, Canada; 2 Department of Health Research Methods, Evidence, and Impact, McMaster University, Hamilton, ON, Canada; 3 Institute of Dentistry, University of Eastern Finland, Kuopio, Finland; 4 Department of Dentistry and Oral Health, Aarhus University, Aarhus, Denmark; 5 Department of Statistics, Statistical Consultation Unit, StaBLab, LMU Munich, Munich, Germany; 6 Topic Group Dental Diagnostics and Digital Dentistry, ITU/WHO Focus Group AI on Health, Berlin, Germany; 7 Department of Oral Diagnostics, Digital Health and Health Services Research, Charité –Universitätsmedizin Berlin, Berlin, Germany; 8 Department of Conservative Dentistry and Oral Health, Riga Stradins University, Riga, Latvia; 9 Faculty of Dentistry, University of Valparaiso, Valparaiso, Chile; 10 Baltic Biomaterials Centre of Excellence, Headquarters at Riga Technical University, Riga, Latvia; 11 Centre for Journalology, Clinical Epidemiology Program, Ottawa Hospital Research Institute, Ottawa, Canada; 12 Faculty of Medicine, School of Epidemiology, Public Health and Preventive Medicine, University of Ottawa, Ottawa, Canada; University of Bristol, UNITED KINGDOM OF GREAT BRITAIN AND NORTHERN IRELAND

## Abstract

**Background:**

According to the FAIR principles (Findable, Accessible, Interoperable, and Reusable), scientific research data should be findable, accessible, interoperable, and reusable. The COVID-19 pandemic has led to massive research activities and an unprecedented number of topical publications in a short time. However, no evaluation has assessed whether this COVID-19-related research data has complied with FAIR principles (or FAIRness).

**Objective:**

Our objective was to investigate the availability of open data in COVID-19-related research and to assess compliance with FAIRness.

**Methods:**

We conducted a comprehensive search and retrieved all open-access articles related to COVID-19 from journals indexed in PubMed, available in the Europe PubMed Central database, published from January 2020 through June 2023, using the *metareadr* package. Using *rtransparent*, a validated automated tool, we identified articles with links to their raw data hosted in a public repository. We then screened the link and included those repositories that included data specifically for their pertaining paper. Subsequently, we automatically assessed the adherence of the repositories to the FAIR principles using FAIRsFAIR Research Data Object Assessment Service (F-UJI) and *rfuji* package. The FAIR scores ranged from 1–22 and had four components. We reported descriptive analysis for each article type, journal category, and repository. We used linear regression models to find the most influential factors on the FAIRness of data.

**Results:**

5,700 URLs were included in the final analysis, sharing their data in a general-purpose repository. The mean (standard deviation, SD) level of compliance with FAIR metrics was 9.4 (4.88). The percentages of moderate or advanced compliance were as follows: Findability: 100.0%, Accessibility: 21.5%, Interoperability: 46.7%, and Reusability: 61.3%. The overall and component-wise monthly trends were consistent over the follow-up. Reviews (9.80, SD = 5.06, n = 160), articles in dental journals (13.67, SD = 3.51, n = 3) and Harvard Dataverse (15.79, SD = 3.65, n = 244) had the highest mean FAIRness scores, whereas letters (7.83, SD = 4.30, n = 55), articles in neuroscience journals (8.16, SD = 3.73, n = 63), and those deposited in GitHub (4.50, SD = 0.13, n = 2,152) showed the lowest scores. Regression models showed that the repository was the most influential factor on FAIRness scores (R^2^ = 0.809).

**Conclusion:**

This paper underscored the potential for improvement across all facets of FAIR principles, specifically emphasizing Interoperability and Reusability in the data shared within general repositories during the COVID-19 pandemic.

## Introduction

The COVID-19 pandemic introduced a significant shift in the scientific publishing ecosystem, catalyzed by the urgency of sharing findings in a rapidly evolving global health crisis [1]. This led to an unprecedented proliferation of preprint publications and open-access materials, allowing researchers worldwide to access peer-reviewed and non-peer-reviewed findings freely [2]. Open-access publications are just one aspect of a larger, comprehensive movement: open science [3]. Some funders and journals, such as CIHR [4], NIH [5], BMJ [6], and PLOS [7], have aligned themselves with open science. Central to open science are three components: open protocols, open-access publications, and open data; collectively, they enhance transparency, collaboration and dissemination [8].

Data openness is the cornerstone of research validation and replication, fortifying scientific credibility [9]. Precise, exhaustive datasets form the bedrock on which scientific conclusions rest and inform the development of further research. In contrast, a major issue during the COVID-19 pandemic was data paucity, which is the lack of high-quality, timely, and reliable data, partially feeding the burgeoning “infodemic” [10] where an excessive amount of information, including false or misleading content, is circulated in digital and physical spaces. Inaccurate or insufficient data deficits can lead to skepticism and mistrust toward research findings, eroding public confidence and impeding a science-informed response [11].

To optimally utilize open research data, it must align with the FAIR principles, i.e., that data are Findable, Accessible, Interoperable, and Reusable [12]. These criteria foster better data utility, extending its applicability beyond the original work and facilitating the exploration of different theories, substantiation of claims, probing of debates, preventing unnecessary duplication, and deriving fresh knowledge from existing data [13]. While privacy concerns may impede complete data openness, sharing metadata can be a partial but meaningful substitute [14]. Metadata can provide insights into the nature of the data and its structure, facilitating interpretation and usability [15].

The FAIR guiding principles have emerged as an important framework for promoting effective data sharing and reuse in scientific research. However, measuring FAIRness and translating these principles into concrete, measurable metrics has proven challenging, requiring close collaboration among research communities to define applicable metrics for different data types and sharing practices. While numerous efforts have attempted to develop FAIR assessment approaches, including both manual evaluations and automated tools like FAIRshake [16] and the FAIR Evaluator [17], significant limitations remain in comprehensively and accurately measuring FAIR implementation. This is particularly true for context-specific and continuous aspects such as interoperability and reusability, which often require subjective assessments within narrow contexts [18, 19]. For instance, the meaning of "rich metadata" (principle F2) can vary in different settings, and the mere presence of a license or use of identifiers does not guarantee data is truly reusable or interoperable in practice.

Although automated tools like F-UJI can monitor the uptake of common base FAIR principles (e.g., considering data "rich" if minimum core citation and descriptive metadata are provided through appropriate fields) [20], they struggle to capture the nuanced nature of these principles fully. Evaluation of the FAIRness of open data on such context-specific detail is not possible automatically and would require subjective assessments and quite a narrow context where all principles would have the same meanings and operationalizations [18]. However, one can monitor the uptake of the common base set of FAIR principles by standardized automated tools. As an example of a common base requirement, shared data would be considered rich if some metadata has been made available via common web standards, and minimum core citation and descriptive metadata are specified (such as creator, title, publication date, publisher, summary, keywords) through appropriate metadata fields [20]. This study builds on previous work to develop more holistic FAIR metrics, while acknowledging the inherent challenges in automating assessments of complex socio-technical issues surrounding data sharing and reuse.

Notably, recent studies demonstrate that data sharing as the first requirement for open data remains sparse in medical research [21], and that shared data often fail to meet the FAIR principles [22]. For assessing research integrity, better decision-making, gaining public trust, and future preparedness, it is essential to clarify the data quality generated throughout the COVID-19 pandemic [23]. Thus, this study aimed to assess the adherence of COVID-19-related research data to the FAIR principles (or FAIRness), a critical step towards improving data quality and trust in scientific outputs.

## Methods

The protocol of this study was deposited on the Open Science Framework (OSF) website (https://doi.org/10.17605/OSF.IO/XAYP9) prior to beginning the study. All the codes and data related to the study were shared via its OSF repository (https://doi.org/10.17605/OSF.IO/YMD6W) and GitHub (https://github.com/choxos/covid-fairness) at the time of submission of the manuscript. Deviations from the protocol are available in S1 Text.

### Data sources and study selection

First, we searched for all open access PubMed-indexed records available in the Europe PubMed Central (EPMC) database from January 1, 2020 (when Chinese authorities announced the new virus), to June 30, 2023, using the *europepmc* package [24] in R [25]. EPMC includes all records available through PubMed and PubMed Central and allows the automatic retrieval of records that were not available through the PubMed website. Since our automated tools were optimized for the English language, only open-access English papers were included. We used the following query to identify all open-access PubMed-indexed papers in English from the beginning of 2020 until April 15, 2023.

To find COVID-19-related papers, we used the LitCovid database (https://ncbi.nlm.nih.gov/research/coronavirus). LitCovid, sponsored by the National Library of Medicine, is a curated literature hub to track up-to-date COVID-19-related scientific information in PubMed. LitCovid is updated daily with newly identified relevant articles organized into curated categories. It uses machine learning and deep learning algorithms [26–28]. We merged our database with the one from LitCovid using inner-join. To do so, we compared PubMed IDs (PMIDs) retrieved from EPMC with the PMIDs in LitCovid. If a PMID was available in both, it was considered an open-access PubMed-indexed COVID-19-related paper.

As we were interested in subgroup analyses along study types, we further used EPMC’s *pubType* column to detect reviews (“review|systematic review|meta-analysis|review-article”), research articles (“research-article”) and letters (“letter”). Since EPMC’s categorization for randomized trials was deemed not to be sensitive enough [29–31] and did not provide any category for all observational studies, we used the L·OVE (Living OVerview of Evidence, https://iloveevidence.com) database to detect randomized trials and observational studies and classify them as such. L·OVE, powered by Epistemonikos Foundation, is an open platform that maps and organizes the best evidence in various medical and health sciences fields [32]. We applied the “Reporting data” filter on the L·OVE website to exclude protocols of trials or observational studies. We used PMIDs of COVID-19-related RCTs and observational studies provided in these downloaded datasets from the L·OVE website to detect RCTs and observational studies in our main dataset of all open-access COVID-19-related papers.

We downloaded all identified open-access COVID-19-related available records in XML full-text format using the *metareadr* package [33] from the EPMC database.

### Data extraction

We used the *rtransparent* package [34] for programmatically assessing data availability in the included studies. The reliability of this package has previously been validated with an accuracy of 94.2% (89.7%–97.9%) in detecting the data availability of assessed papers [35]. The *rtransparent* uses the *oddpub* package [36] for detecting data-sharing statements in XML files of papers. Briefly, the *oddpub* package uses regular expressions to identify whether an article mentions a) a general database in which data are frequently deposited (e.g., figshare); b) a field-specific database in which data are frequently deposited (e.g., dbSNP); c) online repositories in which data/code are frequently deposited (e.g., GitHub); d) language referring to commonly shared file formats (e.g., csv); e) language referring to the availability of data as a supplement (e.g., “supplementary data”); and f) language referring to the presence of a data sharing statement (e.g., “data availability statement”). It finally checks whether these were mentioned in the context of positive statements (e.g., “can be downloaded”) or negative statements (e.g., “not deposited”) to produce its final adjudication. This adjudication indicates whether a data sharing statement is present, which aspect of data sharing was detected (e.g., mention of a public database), and then extracts the phrase in which this was detected.

Our previous study showed low FAIRness of data provided in field-specific databases and supplements [22]. This is due to a lack of some properties that reduce FAIRness, such as the lack of an identifier to the dataset, non-machine-readable metadata, and the use of non-general file formats in field-specific databases. Therefore, we focused on studies that linked to a public database for their data. Another reason was to reduce the burden of work that added little to our study and helped automatize the workload.

After filtering the studies that provided their data in one of the listed general-purpose repositoriesy (limited to the ones that were defined and detected by the *oddpub* package, the list of these repositories is available in S2 Text), we searched for the URL to their dataset in their full-text XML files. To do this, we used keywords related to general-purpose databases and identified every URL that contained one of the keywords. These keywords are available in S1 Appendix. After obtaining all the possible URLs to datasets, we manually screened the links. We included a URL only when it belonged to that specific study, i.e., we excluded URLs to general datasets notably, the COVID-19 Data Repository by Johns Hopkins University, the COVID Chest X-Ray dataset by IEEE, and Covid-19 Data in the United States by New York Times. Comprehensive details of our approach of including URLs are available in S2 Text.

We used the Scimago Journal & Country Rank (SJR, https://www.scimagojr.com) to extract the SJR score, H-index, publisher, subject area, and category of the journals.

### FAIRness assessment

FAIR Principles include four main components about how the shared data/metadata should be: Findable, Accessible, Interoperable, and Reusable [12]. These four components are divided into 10 subcomponents (F1-F4, A1-A2, I1-I3, and R1) and five sub-subcomponents. To measure the level of FAIRness, a tool named FAIRsFAIR Research Data Object Assessment Service (F-UJI) has been developed by the FAIRsFAIR project [20]. F-UJI is a web service to programmatically assess the FAIRness of research data objects based on metrics developed by the FAIRsFAIR project. It checks each component and subcomponents of FAIRness and assigns scores for each metric and an overall score. The lowest score for each component is 0 and the highest ranges from 3–8; the overall score ranges between 1 (because all have URLs, FsF-F1-01D = 1) and 22. The metrics, scores, and definitions of each metric are illustrated in S1 Table.

After finalizing the URLs, we used the F-UJI tool, which is based on Python, to assess the FAIRness of each dataset automatically. We used the rfuji package [33] in R, an application programming interface (API) client for F-UJI. The workflow for running each software is available in S3 Text.

### Analysis

We reported the general characteristics of papers that had shared their data in a general-purpose repository. For the FAIRness assessment, we performed a descriptive analysis of compliance with FAIR metrics. We explored FAIR-level differences between different journals and trends over time. We established a categorization system comprising four compliance levels with FAIR principles for each component of FAIR: 0: incomplete; 1: initial; maximum score: advanced; and every other score between initial and advanced: moderate.

We performed the Kruskal-Wallis rank sum test to compare the level of FAIRness between different article types, journal subject areas, and repositories since the data were skewed. To determine the most influential factor among article type, journal subject area, and repository, we ran different regression models and adjusted for the number of citations and SJR score. Then, we compared the R^2^ of the models. The factor in the model with the highest R^2^ was considered the most influential factor.

## Results

### General characteristics

From January 1, 2020, until April 15, 2023, there were 345,332 COVID-19-related articles, including open-access and non-open-access publications. Of those, 257,348 (74.5%) had available full text from the EPMC. However, 7 (<0.01%) of these open-access articles were not downloadable because of technical issues and were excluded from our analyses. Consequently, the sample included 257,341 full-text articles.

Of these, 20,873 (8.1%) were detected to have shared their data; of these, 8,015 (38.4%) had shared their data in a general-purpose repository. After screening the URLs, 6,180 URLs were included.

**Fig 1:**
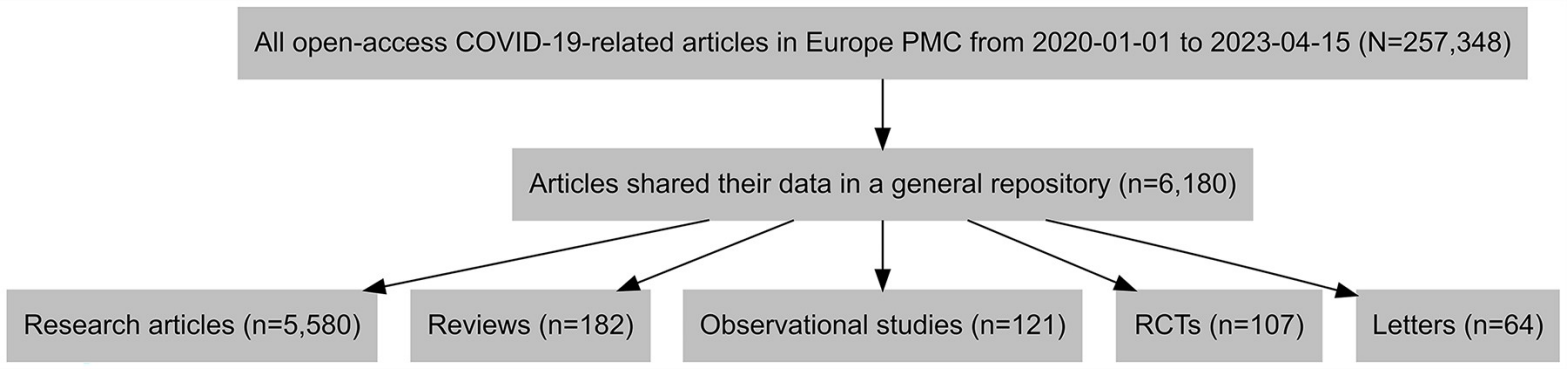
The flow diagram of the study.

Out of those articles which shared their data in a general-purpose repository, 746 (12.1%) were published in 2020, 2,394 (38.7%) in 2021, 2,466 (39.9%) in 2022, and 574 (9.3%) in 2023 (censored data) by April 15. More than 9 in 10 of the papers (n = 5,580, 90.3%) were research articles, followed by reviews (n = 182, 2.9%), observational studies (n = 121, 2.0%), and RCTs (n = 107, 1.7%), and one percent (n = 64) were letters (Fig 1). The papers were from 1,067 different journals, with the top three being PLoS ONE (n = 816, 13.2%), Scientific Reports, (n = 327, 5.3%), and Nature Communications (n = 209, 3.4%).

### FAIRness results

The FAIRness score for 480 repositories was 1, meaning either the repository was inaccessible or had nothing. We eliminated these from our analyses. Therefore, our final analyses were performed on the FAIRness results of 5,700 repositories.

The mean (standard deviation, SD) level of compliance with FAIR metrics was 9.4 (4.88). The mean for each metric was as follows: findability: 4.3 (1.85) of 7; accessibility: 1.2 (0.49) of 3; interoperability: 1.3 (1.30) of 4; and Reusability: 2.6 (1.56) of 8. The compliance by metric is shown in Table 1.

**Table 1:** Summary of FAIR metrics.

Metric	Mean (SD)	Range	Possible scores
**FAIR**	9.4 (4.88)	1.5–19.0	1–22
**F** **indable**	4.3 (1.85)	1.5–7	1–7
*F1*. *(Meta)data are assigned a globally unique and persistent identifier*.	1.5 (0.50)	1–2	1–2
*F2*. *Data are described with rich metadata*.	1.1 (0.66)	0–2	0–2
*F3*. *Metadata clearly and explicitly include the identifier of the data they describe*.	0.2 (0.41)	0–1	0–1
*F4*. *(Meta)data are registered or indexed in a searchable resource*.	1.4 (0.52)	0–2	0–2
**A** **ccessible**	1.2 (0.49)	0–3	0–3
*A1*. *(Meta)data are retrievable by their identifier using a standardized communications protocol*.	1.2 (0.49)	0–3	0–3
**I** **nteroperable**	1.3 (1.30)	0–4	0–4
*I1*. *(Meta)data use a formal*, *accessible*, *shared*, *and broadly applicable language for knowledge representation*.	1.0 (0.92)	0–2	0–2
*I2*. *(Meta)data use vocabularies that follow FAIR principles*.	0.1 (0.24)	0–1	0–1
*I3*. *(Meta)data include qualified references to other (meta)data*.	0.3 (0.45)	0–1	0–1
**R** **eusable**	2.6 (1.56)	0–7	0–8
*R1*. *(Meta)data are richly described with a plurality of accurate and relevant attributes*.	0.7 (0.60)	0–3	0–2
*R1*.*1*. *(Meta)data are released with a clear and accessible data usage license*.	0.8 (0.92)	0–2	0–2
*R1*.*2*. *(Meta)data are associated with detailed provenance*.	1.0 (0.10)	0–1	0–2
*R1*.*3*. *(Meta)data meet domain-relevant community standards*.	0.2 (0.37)	0–1	0–1

**Fig 2:**
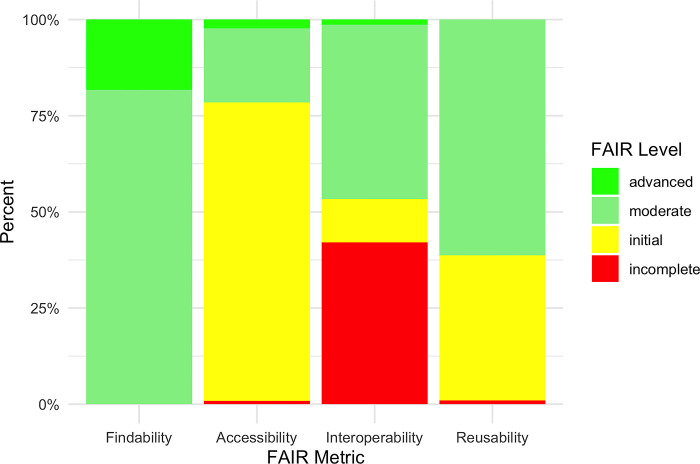
FAIR level by metric (percentage).

The percentages of moderate or advanced compliance were as follows: Findability: 100.0%, Accessibility: 21.5%, Interoperability: 46.7%, and Reusability: 61.3% (Fig 2).

**Fig 3:**
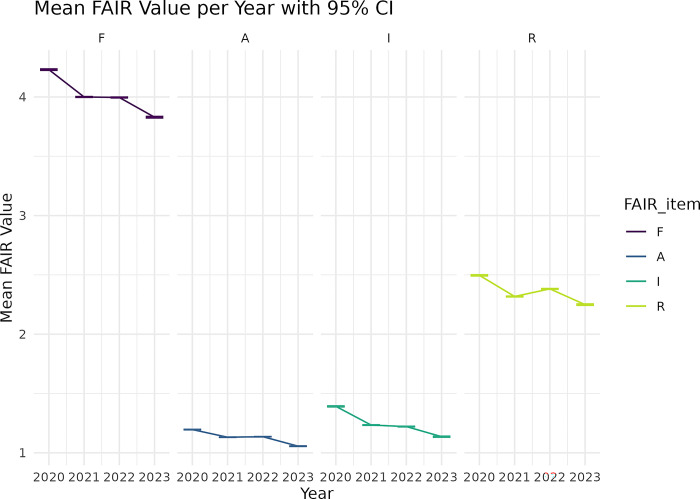
Yearly trend for FAIR components.

Fig 3 shows the annual mean value of FAIRness components. Generally, a downward trend was demonstrated. The monthly trend and annual trend for subcomponents are available in S1 and S2 Figs. The overall and component-wise monthly trends were consistent over the follow-up.

### FAIRness by article type

Reviews had the highest mean FAIRness score (9.80, SD = 5.06, n = 160), whereas research letters had the lowest score (7.83, SD = 4.30, n = 55). The Kruskal-Wallis rank sum test showed a *P*-value of 0.15 for the group differences. Table 2 shows the detailed information.

**Table 2:** FAIRness score for each article type (mean and SD).

Article type	Mean FAIRness score (SD)
Reviews (n = 160)	9.80 (5.06)
Observational studies (n = 115)	9.37 (4.87)
RCTs (n = 90)	9.04 (4.20)
Research letters (n = 55)	7.83 (4.30)
Other research articles (n = 5,280)	9.40 (4.89)
*P*-value	0.15

### FAIRness by journal subject area

Articles in dental journals had the highest mean FAIRness score (13.67, SD = 3.51, n = 3), whereas articles in neuroscience journals had the lowest score (8.16, SD = 3.73, n = 63). The Kruskal-Wallis rank sum test showed a *P*-value of <0.001 for the difference between the groups. Table 3 shows the detailed information.

**Table 3:** FAIRness score for each journal subject area (mean and SD).

Journal area	Mean FAIRness score (SD)
Dentistry (n = 3)	13.67 (3.51)
Immunology (n = 151)	10.40 (5.14)
Biochemistry (n = 811)	10.28 (4.89)
Nursing (n = 11)	10.27 (5.17)
Multidisciplinary (n = 1,527)	9.91 (4.95)
Pharmacology (n = 35)	9.40 (5.47)
Health (n = 580)	9.10 (5.05)
Medicine (n = 492)	9.00 (4.88)
Psychology (n = 368)	8.45 (2.98)
Neuroscience (n = 63)	8.16 (3.73)
Other (n = 1,659)	8.85 (4.97)
*P*-value	<0.001

### FAIRness by repository

Harvard Dataverse had the highest mean FAIRness score (15.79, SD = 3.65, n = 244), whereas GitHub had the lowest score (4.50, SD = 0.13, n = 2,152). The Kruskal-Wallis rank sum test showed a *P*-value of <0.001 for the difference between the groups. Table 4 shows the detailed information.

**Table 4:** FAIRness score for each repository (mean and SD).

Repository	Mean FAIRness score (SD)
Harvard Dataverse (n = 244)	15.79 (3.65)
figshare (n = 740)	14.57 (3.50)
Mendeley (n = 349)	13.90 (1.17)
GigaDB (n = 7)	13.79 (1.95)
Zenodo (n = 897)	13.76 (2.28)
Dryad (n = 140)	13.73 (4.31)
OSF (n = 1,134)	8.43 (2.16)
Dataverse NL (n = 2)	7.00 (7.78)
GitHub (n = 368)	4.50 (0.13)
*P*-value	<0.001

### The most influential factor

The repository’s R2 was the highest (R2 = 0.809), and the model with all these three factors’ R2 was 0.812 (Table 5). The *P*-value for the number of citations and SJR score in all models was above 0.29.

**Table 5:** R^2^ for different linear regression models, adjusted for the number of citations and SJR score.

Model	R^2^
Repository	0.8088
Journal subject area	0.0143
Article type	0.0015
Full	0.8116

## Discussion

Our study aimed to scrutinize FAIR principles adherence in data shared by COVID-19-related articles. We programmatically analyzed the data sharing from 257,348 COVID-19-related articles indexed in PubMed. Of the articles reviewed, 8.1% were programmatically identified to share their data. Of those, 38.4% utilized a general-purpose repository for data sharing. Finally, we identified functioning links to the general-purpose repositories for 6,180 articles, representing 2.4%. In those, the overall average FAIR compliance score was 9.4. Compared to the highest possible score in each component, the largest deficiencies were Reusability, while Findability scored the highest. No considerable changes in the FAIRness compliance were detected over follow-up.

Notable differences in FAIRness compliance emerged based on the repository and journal subject area, but interestingly, they were not based on the type of article. Harvard Dataverse led repository rankings with a mean FAIRness score of 15.8, while GitHub scored the lowest at 4.5. In addition, the regression analysis implied that the repository had the greatest impact on the FAIRness of data shared. These differences likely stem from how FAIR principles have been implemented in the structure and workflows within each repository. For instance, in Harvard Dataverse, the principles are implemented systematically to metadata [37], whereas in GitHub, following FAIR principles is much more up to people sharing their data. For instance, data shared via GitHub lacks DOI, and archiving one’s data and materials in general repositories is recommended [38]. Differences in FAIRness by journal subject area are likely driven by the repository chosen because there were no differences in FAIRness according to the type of article.

As demonstrated through the study results, data sharing in the medical and healthcare research community remains inappropriately low. This suboptimal adherence to the FAIRness principles has several implications for COVID-19 research. First, it makes it difficult for other researchers to find and use the data. Second, it reduces the reproducibility of research findings. Third, it limits the potential for data reuse and collaboration [12, 39, 40]. Similar studies [35, 41] found that only 20% and 18.3% of biomedical articles available in PubMed had a data-sharing statement available in PubMed-based research published between 2015 and 2017 had data available. The absence of transparency in scientific research leads to serious issues with reproducibility, primarily due to the unavailability of data and code. Such opacity significantly hinders a clear understanding of the research methodology and applicability. In the future, it would be important to understand why studies do not share their data and whether they have appropriate reasons to do so. Potentially helping in the advancement of more open and reusable data. However, despite the potential benefits of data sharing, its impact on encouraging peer reanalysis has been minimal so far [42].

Our study showed that the shared data were not generally reusable. Sharing open data does not translate to legal permissions for reuse and redistribution [19]. This is an important issue since without permission to reuse data, collective endeavours to improve science will be impeded. Although data-sharing practices among journals have seen some progress, existing policies often lack detailed guidelines on ensuring optimal data reusability [43].

Despite the privacy constraints inherent in medical and healthcare research, the guiding principle should be to keep data as open as possible yet as closed as necessary [44]. Existing guidelines elucidate various advantages, such as enhancing drug safety and efficacy monitoring. This, in turn, spurs research innovation and facilitates secondary analyses for addressing new scientific questions [45]. Moreover, adhering to open science practices like data and code sharing per FAIR principles boosts public trust and fosters greater public engagement in scientific research, data collection, and research funding [46]. Our study’s finding of a low percentage of articles with openly accessible data and code further underscores the need to restructure incentives for encouraging open scientific practices among researchers. For instance, the Royal Society recommends that “assessment of university research should reward the development of open data on the same scale as journal articles and other publications” [47]. Furthermore, the San Francisco Declaration on Research Assessment recommends that funding agencies, institutions, and publishers consider the significance and influence of all research outputs, encompassing datasets and software when conducting research evaluations [48]. Similarly, the Hong Kong Principles for evaluating researchers endorse sharing data and code as an essential component of the publication process [49]. Universities also have an important role. This is where the next generation of researchers are currently learning. Incorporating more visible training on open science, including the FAIR principles, will likely pay dividends to increasing data and code availability and reuse.

### Strengths and limitations

To the best of the authors’ knowledge, this is the first study to programmatically assess the application of FAIR principles to COVID-19 research since its official announcement by the Chinese government. Given the recent advancements in algorithmic development, our study offers an initial machine evaluation of the FAIRness of COVID-19 research data. Our study also notes several mentionable strengths: utilizing guidelines like the FAIR principles, we offer a comprehensive evaluation of research data generated in the context of the COVID-19 epidemic. From the idea’s inception to the research submission, transparency was maintained. The study protocol was pre-published on the OSF website. After manuscript submission, all relevant codes and data were shared through OSF and GitHub. Our approach also incorporates programmatic detection of data availability and repository [35].

A limitation of our study is that the study sample focuses solely on articles available from the European PMC database, representing 74.5% of all COVID-19-related medical publications indexed in Pubmed. Moreover, the algorithms from the *rtransparent* package in R were developed before the pandemic, potentially affecting their accuracy in detecting data sharing compared to studies on topics preceding the COVID-19 outbreak. We also only investigated data shared via general repositories, so our findings are directly generalizable to studies sharing data, e.g., as supplementary material on the journal’s website, which is likely not generalizable to all COVID-19- related research because of existing domain-specific repositories (such as the COVID-19 Data Repository, https://www.openicpsr.org/openicpsr/covid19). We also did not evaluate databases that store and manage sensitive patient data (or other protected data), because such databases cannot be evaluated with the available automated tools.

The limitation of the automated approach using common base requirements of FAIR is that it falls short in assessing FAIRness in detail. For instance, assessing data licensing and its implications for data reuse is far more nuanced [19] than what is included in the F-UJI tool, which detects whether metadata contains a standard, machine-readable license, and the information represented using an appropriate metadata element [20]. In the future, a more detailed investigation of data FAIRness in the context in which a dataset could be utilized should be performed and not just whether it follows the base requirements of FAIR principles investigated here.

Our results highlight both the progress and ongoing challenges in developing robust, automated metrics for assessing FAIR implementation. While our approach was able to efficiently evaluate certain aspects like findability and technical accessibility across a large number of datasets, significant gaps remain in comprehensively measuring interoperability and reusability through automated means. As noted by Carbon et al. [19], critical factors like licensing terms, semantic interoperability, and privacy considerations for sensitive data are often overlooked or inadequately captured by existing FAIR metrics. The limitations of our automated assessment underscore the need for complementary manual evaluation and expert curation to provide a more complete picture of FAIRness.

For example, our tool was able to detect the presence of persistent identifiers and ontology terms, but could not assess whether these were implemented in a way that truly enhanced interoperability. Issues like identifier hygiene and the proliferation of redundant identifiers require human judgment to evaluate properly. Similarly, while we could check for the existence of licensing information, determining if those terms actually enable reuse often requires legal expertise. The context-specific nature of interoperability and reusability poses a major challenge for developing universal metrics.

Moving forward, FAIR assessment approaches will likely need to balance automated methods with targeted manual evaluation to address these nuanced issues. There may be opportunities to enhance automated tools with more sophisticated natural language processing and machine learning techniques to better parse licensing terms and assess semantic interoperability. However, some degree of human oversight and field-specific expertise will likely remain necessary, particularly for evaluating sensitive datasets with complex governance requirements. Ultimately, refining FAIR metrics and assessment methods remains an active area of research requiring ongoing collaboration between data providers, users, and governance experts.

## Conclusions

Our findings highlight room for improvement in all basic components of FAIR, particularly in terms of Interoperability and Reusability, in the data shared in general repositories during the COVID-19 pandemic. First, more data should be shared, but its FAIRness could also be improved. For instance, data FAIRness could be considered when formulating new journal data-sharing policies. Joint efforts involving all stakeholders in scientific publishing—researchers, editors, publishers, and funders—accompanied by data repositories—are very welcome.

Our study also highlighted some challenges of large-scale automatic assessment of data FAIRness. First, more widespread automatic linking of research and shared data would be needed for larger-scale automatic analyses. On the other hand, solutions to evaluate data FAIRness in enough detail and automatic evaluation of data in protected repositories would also be highly needed.

Enhanced availability of high-quality open research data would bolster confidence in scientific findings and interpretations, narrowing the information gap among researchers, clinicians, and the general populace. Utilizing FAIR principles would facilitate human and machine accessibility to research data, augmenting the efficacy of tools designed to navigate intricate, multi-dimensional health-related data. Such machine-actionable data, offering real-time insights, would fortify the emergence of data-driven medicine and ultimately advance healthcare research goals, thereby elevating overall health and quality of life.

## Supporting information

S1 Text Deviations from the protocol. (DOCX)

S2 Text Our approach for screening the URLs. (DOCX)

S3 Text The workflow for running F-UJI and RF-UJI in Windows. (DOCX)

S1 Appendix Keywords to identify general-purpose databases. (CSV)

S1 Table The FAIR metrics, scores, and definitions of each metric. (DOCX)

S1 Fig Monthly trend for FAIR and its subcomponents. (TIFF)

S2 Fig Yearly trend for subcomponents of FAIR. (TIFF)

## References

[1] Brainard J. No revolution: COVID-19 boosted open access, but preprints are only a fraction of pandemic papers. Science [Internet]. 2021 Sep 8 [cited 2023 Sep 10]; Available from: https://www.science.org/content/article/no-revolution-covid-19-boosted-open-access-preprints-are-only-fraction-pandemic-papers

[2] Watson C. Rise of the preprint: how rapid data sharing during COVID-19 has changed science forever. Nat Med. 2022 Jan;28(1):2–5. doi: 10.1038/s41591-021-01654-6 35031791

[3] McKiernan EC, Bourne PE, Brown CT, Buck S, Kenall A, Lin J, et al. How open science helps researchers succeed. eLife. 2016 Jul 7;5:e16800. doi: 10.7554/eLife.16800 27387362 PMC4973366

[4] Canadian Institutes of Health Research. Health Research Data: Strategies and policies [Internet]. Canadian Institutes of Health Research; 2021 [cited 2023 Oct 6]. Available from: https://cihr-irsc.gc.ca/e/49940.html

[5] National Institutes of Health. Data Management and Sharing Policy [Internet]. National Institutes of Health; [cited 2023 Oct 6]. Available from: https://sharing.nih.gov/data-management-and-sharing-policy

[6] British Medical Journal. Data sharing [Internet]. British Medical Journal; [cited 2023 Oct 6]. Available from: https://authors.bmj.com/policies/data-sharing/

[7] Public Library of Science. Data Availability [Internet]. Public Library of Science; [cited 2023 Oct 6]. Available from: https://journals.plos.org/plosone/s/data-availability

[8] Raittio E, Sofi‐Mahmudi A, Uribe SE. Research transparency in dental research: A programmatic analysis. Eur J Oral Sci. 2023 Feb;131(1):e12908. doi: 10.1111/eos.12908 36482006 PMC10108147

[9] Miyakawa T. No raw data, no science: another possible source of the reproducibility crisis. Mol Brain. 2020 Dec;13(1):24, s13041-020-0552–2. doi: 10.1186/s13041-020-0552-2 32079532 PMC7033918

[10] World Health Organization. Managing the COVID-19 infodemic: Promoting healthy behaviours and mitigating the harm from misinformation and disinformation. 2020;

[11] Bromme R, Mede NG, Thomm E, Kremer B, Ziegler R. An anchor in troubled times: Trust in science before and within the COVID-19 pandemic. Gesser-Edelsburg A, editor. PLOS ONE. 2022 Feb 9;17(2):e0262823. doi: 10.1371/journal.pone.0262823 35139103 PMC8827432

[12] Wilkinson MD, Dumontier M, Aalbersberg IjJ, Appleton G, Axton M, Baak A, et al. The FAIR Guiding Principles for scientific data management and stewardship. Sci Data. 2016 Dec;3(1):160018. doi: 10.1038/sdata.2016.18 26978244 PMC4792175

[13] Locher C, Le Goff G, Le Louarn A, Mansmann U, Naudet F. Making data sharing the norm in medical research. BMJ. 2023 Jul 11;p1434. doi: 10.1136/bmj.p1434 37433610

[14] De Kok JWTM, De La Hoz MÁA, De Jong Y, Brokke V, Elbers PWG, Thoral P, et al. A guide to sharing open healthcare data under the General Data Protection Regulation. Sci Data. 2023 Jun 24;10(1):404. doi: 10.1038/s41597-023-02256-2 37355751 PMC10290652

[15] Mons B, Neylon C, Velterop J, Dumontier M, Da Silva Santos LOB, Wilkinson MD. Cloudy, increasingly FAIR; revisiting the FAIR Data guiding principles for the European Open Science Cloud. Inf Serv Use. 2017 Mar 7;37(1):49–56.

[16] Clarke DJB, Wang L, Jones A, Wojciechowicz ML, Torre D, Jagodnik KM, et al. FAIRshake: Toolkit to Evaluate the FAIRness of Research Digital Resources. Cell Syst. 2019 Nov;9(5):417–21. doi: 10.1016/j.cels.2019.09.011 31677972 PMC7316196

[17] Wilkinson MD, Dumontier M, Sansone SA, Bonino Da Silva Santos LO, Prieto M, Batista D, et al. Evaluating FAIR maturity through a scalable, automated, community-governed framework. Sci Data. 2019 Sep 20;6(1):174. doi: 10.1038/s41597-019-0184-5 31541130 PMC6754447

[18] Commission E, Research DG for, Innovation. Turning FAIR into reality–Final report and action plan from the European Commission expert group on FAIR data. Publications Office; 2018.

[19] Carbon S, Champieux R, McMurry JA, Winfree L, Wyatt LR, Haendel MA. An analysis and metric of reusable data licensing practices for biomedical resources. Mehmood R, editor. PLOS ONE. 2019 Mar 27;14(3):e0213090. 10.1371/journal.pone.0213090 PMC6436688 30917137

[20] Devaraju A, Huber R. An automated solution for measuring the progress toward FAIR research data. Patterns. 2021 Nov;2(11):100370. doi: 10.1016/j.patter.2021.100370 34820651 PMC8600246

[21] Hamilton DG, Hong K, Fraser H, Rowhani-Farid A, Fidler F, Page MJ. Prevalence and predictors of data and code sharing in the medical and health sciences: systematic review with meta-analysis of individual participant data. BMJ. 2023 Jul 11;e075767. doi: 10.1136/bmj-2023-075767 37433624 PMC10334349

[22] Uribe SE, Sofi-Mahmudi A, Raittio E, Maldupa I, Vilne B. Dental Research Data Availability and Quality According to the FAIR Principles. J Dent Res. 2022 Jun 2;00220345221101321. 10.1177/00220345221101321 PMC9516597 35656591

[23] Austin CC, Bernier A, Bezuidenhout L, Bicarregui J, Biro T, Cambon-Thomsen A, et al. Fostering global data sharing: highlighting the recommendations of the Research Data Alliance COVID-19 working group. Wellcome Open Res. 2021 May 26;5:267. doi: 10.12688/wellcomeopenres.16378.2 33501381 PMC7808050

[24] Jahn N. europepmc: R Interface to the Europe PubMed Central RESTful Web Service [Internet]. 2021. Available from: https://CRAN.R-project.org/package=europepmc

[25] R Core Team. R: A Language and Environment for Statistical Computing [Internet]. Vienna, Austria: R Foundation for Statistical Computing; 2023. Available from: https://www.R-project.org/

[26] Chen Q, Allot A, Lu Z. Keep up with the latest coronavirus research. Nature. 2020 Mar 12;579(7798):193–193. doi: 10.1038/d41586-020-00694-1 32157233

[27] Chen Q, Allot A, Lu Z. LitCovid: an open database of COVID-19 literature. Nucleic Acids Res. 2021 Jan 8;49(D1):D1534–40. doi: 10.1093/nar/gkaa952 33166392 PMC7778958

[28] Chen Q, Allot A, Leaman R, Wei CH, Aghaarabi E, Guerrerio JJ, et al. LitCovid in 2022: an information resource for the COVID-19 literature. Nucleic Acids Res. 2023 Jan 6;51(D1):D1512–8. doi: 10.1093/nar/gkac1005 36350613 PMC9825538

[29] Wieland LS, Robinson KA, Dickersin K. Understanding why evidence from randomised clinical trials may not be retrieved from Medline: comparison of indexed and non-indexed records. BMJ. 2012 Jan 3;344:d7501. doi: 10.1136/bmj.d7501 22214757

[30] Cohen AM, Smalheiser NR, McDonagh MS, Yu C, Adams CE, Davis JM, et al. Automated confidence ranked classification of randomized controlled trial articles: an aid to evidence-based medicine. J Am Med Inform Assoc JAMIA. 2015 May;22(3):707–17. doi: 10.1093/jamia/ocu025 25656516 PMC4457112

[31] Edinger T, Cohen AM. A large-scale analysis of the reasons given for excluding articles that are retrieved by literature search during systematic review. AMIA Annu Symp Proc AMIA Symp. 2013;2013:379–87. 24551345 PMC3900186

[32] Verdugo-Paiva F, Vergara C, Ávila C, Castro-Guevara JA, Cid J, Contreras V, et al. COVID-19 Living OVerview of Evidence repository is highly comprehensive and can be used as a single source for COVID-19 studies. J Clin Epidemiol. 2022 May;S0895435622001172. doi: 10.1016/j.jclinepi.2022.05.001 35597369 PMC9116966

[33] Serghiou S. metareadr: Downloads data often needed for meta-research. 2022; Available from: https://github.com/serghiou/metareadr

[34] Serghiou S. rtransparent: Identifies indicators of transparency. 2021; Available from: http://github.com/serghiou/rtransparent

[35] Serghiou S, Contopoulos-Ioannidis DG, Boyack KW, Riedel N, Wallach JD, Ioannidis JPA. Assessment of transparency indicators across the biomedical literature: How open is open? Bero L, editor. PLOS Biol. 2021 Mar 1;19(3):e3001107. 33647013 10.1371/journal.pbio.3001107 PMC7951980

[36] Riedel N. oddpub: Detection of Open Data & Open Code statements in biomedical publications [Internet]. 2019. Available from: https://github.com/quest-bih/oddpub

[37] Crosas M. The FAIR Guiding Principles: Implementation in Dataverse [Internet]. 2019 Mar 22 [cited 2023 Sep 14]; MIT. Available from: https://scholar.harvard.edu/mercecrosas/presentations/fair-guiding-principles-implementation-dataverse

[38] GitHub. Referencing and citing content [Internet]. GitHub; [cited 2023 Sep 14]. Available from: https://docs.github.com/en/repositories/archiving-a-github-repository/referencing-and-citing-content

[39] Sofi-Mahmudi A, Raittio E. Transparency of COVID-19-Related Research in Dental Journals. Front Oral Health. 2022 Apr 6;3:871033. doi: 10.3389/froh.2022.871033 35464778 PMC9019132

[40] Sofi-Mahmudi A, Raittio E, Uribe SE. Transparency of COVID-19-related research: A meta-research study. Lucas-Dominguez R, editor. PLOS ONE. 2023 Jul 26;18(7):e0288406. 10.1371/journal.pone.0288406 PMC10370694 37494359

[41] Wallach JD, Boyack KW, Ioannidis JPA. Reproducible research practices, transparency, and open access data in the biomedical literature, 2015–2017. Dirnagl U, editor. PLOS Biol. 2018 Nov 20;16(11):e2006930. doi: 10.1371/journal.pbio.2006930 30457984 PMC6245499

[42] Vazquez E, Gouraud H, Naudet F, Gross CP, Krumholz HM, Ross JS, et al. Characteristics of available studies and dissemination of research using major clinical data sharing platforms. Clin Trials. 2021 Dec;18(6):657–66. doi: 10.1177/17407745211038524 34407656 PMC8595516

[43] Vasilevsky NA, Minnier J, Haendel MA, Champieux RE. Reproducible and reusable research: are journal data sharing policies meeting the mark? PeerJ. 2017 Apr 25;5:e3208. doi: 10.7717/peerj.3208 28462024 PMC5407277

[44] Landi A, Thompson M, Giannuzzi V, Bonifazi F, Labastida I, Da Silva Santos LOB, et al. The “A” of FAIR–As Open as Possible, as Closed as Necessary. Data Intell. 2020 Jan;2(1–2):47–55.

[45] Mello MM, Francer JK, Wilenzick M, Teden P, Bierer BE, Barnes M. Preparing for Responsible Sharing of Clinical Trial Data. Hamel MB, editor. N Engl J Med. 2013 Oct 24;369(17):1651–8. 24144394 10.1056/NEJMhle1309073

[46] OECD. Open Science [Internet]. Organisation for Economic Co-operation and Development (OECD); 2018 [cited 2023 Oct 14]. Available from: https://www.oecd.org/sti/inno/open-science.htm

[47] The Royal Society Science Policy Centre. Science as an open enterprise [Internet]. The Royal Society; 2012 [cited 2023 Sep 14]. Available from: https://royalsociety.org/topics-policy/projects/science-public-enterprise/report/

[48] The Declaration on Research Assessment (DORA). San Francisco Declaration on Research Assessment [Internet]. The Declaration on Research Assessment (DORA); [cited 2023 Oct 6]. Available from: https://sfdora.org/read/

[49] Moher D, Bouter L, Kleinert S, Glasziou P, Sham MH, Barbour V, et al. The Hong Kong Principles for assessing researchers: Fostering research integrity. PLOS Biol. 2020 Jul 16;18(7):e3000737. doi: 10.1371/journal.pbio.3000737 32673304 PMC7365391

