# Supplementary material for: COVID-19-related research data availability and quality according to the FAIR principles: A meta-research study
Source: PLoS One. 2024 Nov 18;19(11):e0313991. doi: 10.1371/journal.pone.0313991 (PMC11573139; doi:10.1371/journal.pone.0313991)
Supplement: S1 Text — (DOCX) [file pone.0313991.s001.docx]

**S1 Text.** Deviations from the protocol.

In the protocol, we did not specify regression analyses to determine the most influential factor (among article type, journal area, and repository).
