# Supplementary material for: COVID-19-related research data availability and quality according to the FAIR principles: A meta-research study
Source: PLoS One. 2024 Nov 18;19(11):e0313991. doi: 10.1371/journal.pone.0313991 (PMC11573139; doi:10.1371/journal.pone.0313991)
Supplement: S2 Text — (DOCX) [file pone.0313991.s002.docx]

**S2 Text.** Our approach for screening the URLs.

- Each of us has 2656 rows to check.
- The first four rows are from rtransparent. You can see the right URL in the open_data_statements column. If you are still in doubt, please confirm the URL by opening it in your browser.
- In case of having two links to GitHub and another repo, please use the link to the other repo because GitHub is not generally considered a place for data sharing. If no other repo is used, then use the GitHub link.
- If there is no link and just a mention of the name of the repo (like “figshare” and no links to it), it is very probable that there is no link to that repo in the text and it’s just a mentioning. To be sure, you can either check it by open_data_statements or the full text of the article.
- Please only include **one** URL per study.
- Please be careful with any extra characters at the end of the URL, especially **dots**.
- The repos and their doi general form are as follows:
  - figshare: 10.6084
    - Example: <https://doi.org/10.6084/m9.figshare.22132067.v1>
    - Last part is: v1. Many times there is no .v1 at the end and finishes with a number, like here which is 22132067.
  - Dryad: 10.5061
    - Example: <https://doi.org/10.5061/dryad.547d7wmbk>
    - Finishes with some letters. I am not sure if it can finish with numbers.
  - Zenodo: 10.5281
    - Example: <https://doi.org/10.5281/zenodo.5018779>
    - Finishes with a number.
  - Dataverse: 10.7910
    - Example: <https://doi.org/10.7910/dvn/7wfyct>
    - Finishes with letters,
  - DataverseNL: 10.34894
    - Example: <https://doi.org/10.34894/o7mzg8>
    - Finishes with letters and numbers.
  - OSF: 10.17605
    - Example: <https://doi.org/10.17605/osf.io/t5rxb>
    - Finishes with letters and numbers.
  - Mendeley: 10.17632
    - Example: <http://dx.doi.org/10.17632/x7h23bvdbr>
    - Finishes with letters and numbers.
  - GigaDB: 10.5524
    - Example: <http://doi.org/10.5524/102336>
    - Finishes with numbers.
  - OpenNeuro: 10.18112
    - Example: <https://doi.org/10.18112/openneuro.ds001461.v1.0.3>
    - Finishes with the version number.
- Some may not report doi and just put the generic URL of the repo. These cases should also be included. The URL pattern for each is as follows:
  - figshare: [https://figshare.com/articles/dataset/[The](https://figshare.com/articles/dataset/%5BThe) rest of URL]
  - Dryad: [https://datadryad.org/stash/dataset/doi:[DOI](https://datadryad.org/stash/dataset/doi:%5BDOI) will be here]
  - Zenodo: [https://zenodo.org/record/[the](https://zenodo.org/record/%5Bthe) last part of DOI]
  - Dataverse: [https://dataverse.harvard.edu/dataset.xhtml?persistentId=doi:[DOI](https://dataverse.harvard.edu/dataset.xhtml?persistentId=doi:%5BDOI) will be here]
  - DataverseNL: [https://dataverse.nl/dataset.xhtml?persistentId=doi:[DOI](https://dataverse.nl/dataset.xhtml?persistentId=doi:%5BDOI) will be here]
  - OSF: [https://osf.io/[special](https://osf.io/%5Bspecial) identifier for the database, letters and numbers]
  - Mendeley: [https://data.mendeley.com/datasets/[the](https://data.mendeley.com/datasets/%5Bthe) second part of DOI will be here]
  - GigaDB: [http://gigadb.org/dataset/[the](http://gigadb.org/dataset/%5Bthe) second part of DOI will be here]
  - OpenNeuro: [https://openneuro.org/datasets/[the](https://openneuro.org/datasets/%5Bthe) part of DOI that starts with ds]
  - GitHub: <https://github.com/username/repo>
