# Supplementary material for: COVID-19-related research data availability and quality according to the FAIR principles: A meta-research study
Source: PLoS One. 2024 Nov 18;19(11):e0313991. doi: 10.1371/journal.pone.0313991 (PMC11573139; doi:10.1371/journal.pone.0313991)
Supplement: S3 Text — (DOCX) [file pone.0313991.s003.docx]

**S5 Text.** The workflow for running F-UJI and RF-UJI in Windows.

1. Install Python.
2. Install Build Tools for Visual Studio 2022: <https://visualstudio.microsoft.com/downloads/>.
3. Install F-UJI: <https://github.com/pangaea-data-publisher/fuji>.
4. Don’t forget to set user details in the configuration file: fuji_server/config/users.py.
5. Run F-UJI in localhost.
6. Install and load RF-UJI in R: <https://github.com/NFDI4Chem/rfuji>.
7. Use this for catching FAIRness results: <https://github.com/NFDI4Chem/rfuji/blob/main/docs/FAIRObjectApi.md#AssessById>.
