# Supplementary material for: COVID-19-related research data availability and quality according to the FAIR principles: A meta-research study
Source: PLoS One. 2024 Nov 18;19(11):e0313991. doi: 10.1371/journal.pone.0313991 (PMC11573139; doi:10.1371/journal.pone.0313991)
Supplement: S1 Table — (DOCX) [file pone.0313991.s005.docx]

**S4 Table.** The FAIR metrics, scores, and definitions of each metric.

| **Component** | **Sub** | **Sub-sub** | **FsF metric** | **Score** | **FsF Definition** | **GO FAIR definition** |
| --- | --- | --- | --- | --- | --- | --- |
| **Findable** | F1 | - | FsF-F1-01D | 1 | Data is assigned a globally unique identifier. | (Meta)data are assigned a globally unique and persistent identifier. |
|  |  | - | FsF-F1-02D | 1 | Data is assigned a persistent identifier. |  |
|  | F2 | - | FsF-F2-01M | 2 | Metadata includes descriptive core elements (creator, title, data identifier, publisher, publication date, summary and keywords) to support data findability. | Data are described with rich metadata (defined by R1 below). |
|  | F3 | - | FsF-F3-01M | 1 | Metadata includes the identifier of the data it describes. | Metadata clearly and explicitly include the identifier of the data they  describe. |
|  | F4 | - | FsF-F4-01M | 2 | Metadata is offered in such a way that it can be retrieved by machines. | (Meta)data are registered or indexed in a searchable resource. |
| **Accessible** | A1 | - | FsF-A1-01M | 1 | Metadata contains the access level and access conditions of the data. | (Meta)data are retrievable by their identifier using a standardized communications protocol. |
|  |  | - | FsF-A1-02M | 1 | Metadata is accessible through a standardized communication protocol. |  |
|  |  | - | FsF-A1-03D | 1 | Data is accessible through a standardized communication protocol. |  |
|  | A1 | A1.1 | -^1^ | - | - | The protocol is open, free, and universally implementable. |
|  |  | A1.2 | -^1^ | - | - | The protocol allows for an authentication and authorization procedure, where necessary. |
|  | A2 | - | FsF-A2-01M | -^2^ | Metadata remains available, even if the data is no longer available. | Metadata are accessible, even when the data are no longer available. |
| **Interoperable** | I1 | - | FsF-I1-01M | 2 | Metadata is represented using a formal knowledge representation language. | (Meta)data use a formal, accessible, shared, and broadly applicable language for knowledge representation. |
|  | I2 | - | FsF-I2-01M | 1 | Metadata uses semantic resources. | (Meta)data use vocabularies that follow FAIR principles. |
|  | I3 | - | FsF-I3-01M | 1 | Metadata includes links between the data and its related entities. | (Meta)data include qualified references to other (meta)data. |
| **Reusable** | R1 | - | FsF-R1-01MD | 2 | Metadata specifies the content of the data. | (Meta)data are richly described with a plurality of accurate and relevant attributes. |
|  | R1 | R1.1 | FsF-R1.1-01M | 2 | Metadata includes license information under which data can be reused. | (Meta)data are released with a clear and accessible data usage license. |
|  |  | R1.2 | FsF-R1.2-01M | 2 | Metadata includes provenance information about data creation or generation. | (Meta)data are associated with detailed provenance. |
|  |  | R1.3 | FsF-R1.3-01M | 1 | Metadata follows a standard recommended by the target research community of the data. | (Meta)data meet domain-relevant community standards. |
|  |  |  | FsF-R1.3-02D | 1 | Data is available in a file format recommended by the target research community. |  |

^1^ There is no FsF metric for A1.1 and A1.2.

^2^ This can only be tested if the object is deleted or replaced. So this test is only applicable for deleted, replaced or obsolete objects. Therefore, FsF has excluded its assessment details from this specification.
