# Supplementary figures and images for: COVID-19-related research data availability and quality according to the FAIR principles: A meta-research study
Source: PLoS One. 2024 Nov 18;19(11):e0313991. doi: 10.1371/journal.pone.0313991 (PMC11573139; doi:10.1371/journal.pone.0313991)

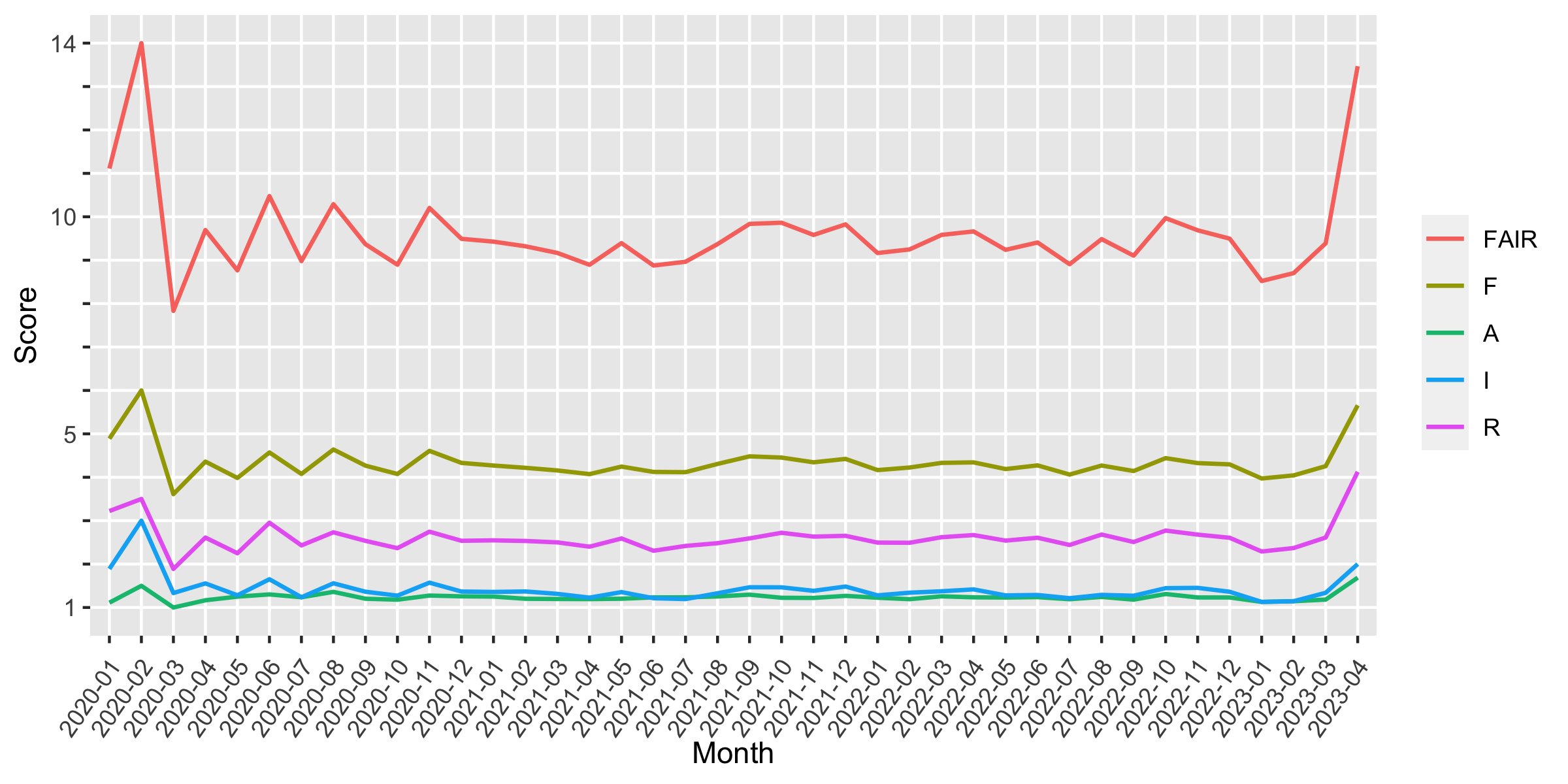

Supplement: S1 Fig — (TIFF) [file pone.0313991.s006.tiff]

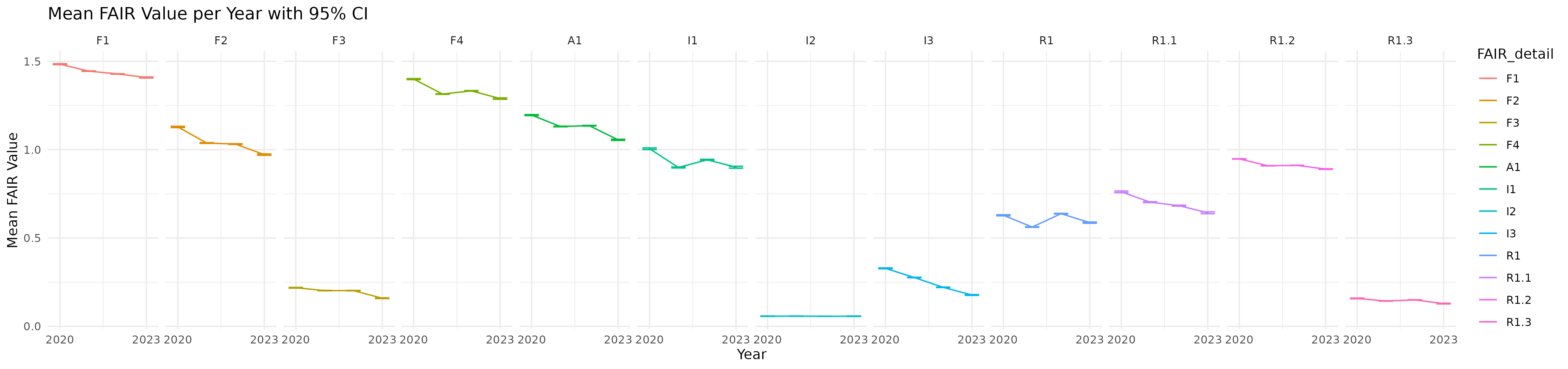

Supplement: S2 Fig — (TIFF) [file pone.0313991.s007.tiff]
